# Clouded leopard phylogeny revisited: support for species recognition and population division between Borneo and Sumatra

**DOI:** 10.1186/1742-9994-4-15

**Published:** 2007-05-29

**Authors:** Andreas Wilting, Valerie A Buckley-Beason, Heike Feldhaar, Jürgen Gadau, Stephen J O'Brien, K Eduard Linsenmair

**Affiliations:** 1 University of Würzburg, Biocentre, Department of Animal Ecology and Tropical Biology, 97074 Würzburg, Germany; 2 Laboratory of Genomic Diversity, National Cancer Institute, Frederick, Maryland, USA; 3 University of Würzburg, Biocentre, Department of Behavioural Physiology and Sociobiology, 97074 Würzburg, Germany; 4 School of Life Science, Arizona State University, Tempe, AZ 85287, USA

## Abstract

**Background:**

The clouded leopard (*Neofelis nebulosa*) is one of the least known cat species and depletion of their forested habitats puts it under heavy pressure. Recently reclassification of Bornean clouded leopards (*N. nebulosa diardi*) to species level (*N.diardi*) was suggested based on molecular and morphological evidence. Since the genetic results were based solely on three Bornean samples we re-evaluated this partition using additional samples of Bornean clouded leopards (N = 7) and we were also able to include specimens from Sumatra (N = 3), which were lacking in previous analysis.

**Results:**

We found strong support for the distinction between *N. nebulosa *and *N. diardi *based on three fragments of mtDNA (900 bp) and 18 microsatellites. Forty-one fixed mitochondrial nucleotide differences and non-overlapping allele sizes in 8 of 18 microsatellite loci distinguished *N. nebulosa *and *N. diardi*. This is equivalent to the genetic divergence among recognized species in the genus *Panthera*. Sumatran clouded leopards clustered with specimens from Borneo, suggesting that Sumatran individuals also belong to *N. diardi*. Additionally, a significant population subdivision was apparent among *N. diardi *from Sumatra and Borneo based on mtDNA and microsatellite data.

**Conclusion:**

Referring to their origin on two Sunda Islands we propose to give *N. diardi *the common name "Sundaland clouded leopard". The reduced gene flow between Borneo and Sumatra might suggest the recognition of two subspecies of *N. diardi*. Based on this reclassification of clouded leopards not only species, but also the populations on Borneo and Sumatra should be managed separately and a higher priority should be placed to protect the different populations from extinction.

## Background

Clouded leopards have drawn less attention in the past compared to other species of the *Panthera *lineage. This medium-sized cat (11–25 kg) primarily inhabits densely vegetated habitats and remote areas in South-East Asian subtropical and tropical forests [1,2]. As a consequence of their secretive behaviour [3,4] very little is known about their behaviour and status in the wild and very few studies have focused on this species [5-7]. Likewise, their taxonomy and phylogenetic status remained obscure for a long time [8-11]. Phylogenetic studies consistently reveal that clouded leopards separated from the *Panthera *lineage approximately 6 million years ago (MYA) [12-14]. Only recently the phylogenetic relationships among clouded leopards were assessed [15]. Based on mtDNA, nuclear DNA sequences, microsatellites, fixed chromosomal differences and morphological variation, the reclassification of Bornean clouded leopards (*N. nebulosa diardi*) to species level *N. diardi *was suggested [15,16]. The small sample size of only three Bornean samples in the phylogenetic study reduces confidence in this reclassification of *N. diardi*. Therefore, we present here the phylogenetic analysis with additional individuals from Borneo. Furthermore, we examine the phylogenetic relationships among clouded leopards more closely by including Sumatran samples. Sumatran individuals are necessary for the recognition of a new species on the Sunda Islands, but until now have not been investigated genetically. The goal of this study is to provide a better understanding of the systematic classification of one of the most threatened cat species in Asia. This classification is of utmost importance for conservation and management purposes [17].

## Results

### Mitochondrial DNA analysis

**Figure 1:**
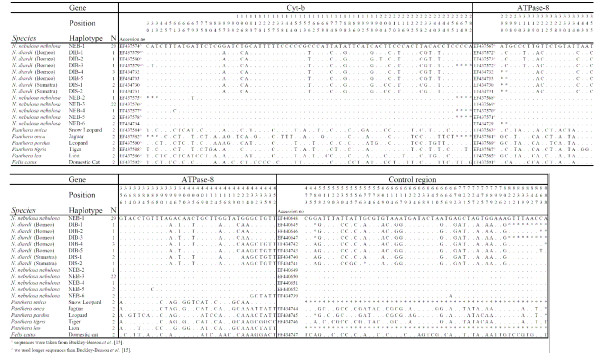
**Table 2: Haplotypes and variable sites in combined analysis of 900 bp of mtDNA sequences**.

Analysis of the phylogenetic relationship among clouded leopards based on a concatenated fragment of 900 bp of mitochondrial DNA comprising sequences from three mitochondrial gene segments (185 bp *ATPase-8*, 286 bp *Cyt b *and 426 bp control region) revealed 54 variable sites among clouded leopards. Of those 39 were fixed nucleotide differences between specimens from the islands of Borneo/Sumatra (*N. diardi*) and specimens from the mainland (*N. nebulosa nebulosa*). There were 12 nucleotide differences in the *Cyt-b *gene (4.2 %), 10 in the *ATPase-8 *gene (5.4 %) and 17 in the control region (3.9 %). In comparison, three *Panthera *species (*P. pardus*, *P.tigris *and *P. onca*) were separated by 38–52 nucleotide differences in the same fragment. Four fixed nucleotide differences distinguished the individuals from Sumatra and Borneo. Altogether we found 13 haplotypes among all the clouded leopards examined. *N. nebulosa nebulosa *(N = 59 GenBank accession no. in Table 2. see Figure 1) had six haplotypes (NEB 1–6), *N. diardi *(Borneo, N = 7) had 5 haplotypes (DIB 1–5) and in *N. diardi *(Sumatra, N = 3) we found 2 haplotypes (DIS 1 & 2).

**Table 1:** Specimens sampled in this study

Code^a^	mtDNA Haplotype^b^	Museum or Zoo ID	Sex^c^	Source	Scientific name	Locale of origin	Birth status^d^	Origin contact
NDB1	DIB4	-	M	fecal	*N. nebulosa diardi*	Sabah, Borneo	W	Sabah Wildlife Department, Kota Kinabalu, Malaysia
NDB2	DIB4	NH 1433	M	hide	*N. nebulosa diardi*	Sabah, Borneo	W	Sabah Museum, Kota Kinabalu, Malaysia
NDB3	DIB4	-	UNK	fecal	*N. nebulosa diardi*	Sabah, Borneo	W	Sabah Wildlife Department, Kota Kinabalu, Malaysia
NDB4	DIB5	SP (P) 256	F	hide	*N. nebulosa diardi*	Sabah, Borneo	W	Sabah Parks, Kinabalu Park, Malaysia
NDS1	DIS1	15470	UNK	dry-tissue	*N. nebulosa diardi*	Sumatra	W	Forschungsinstitut & Naturmuseum Senckenberg, Germany
NDS2	DIS2	1973/269	UNK	dry-tissue	*N. nebulosa diardi*	Northern Sumatra	W	Zoologische Staatssammlung München, Germany
NDS3	DIS2	1973/55	UNK	dry-tissue	*N.nebulosa diardi*	Sumatra	W	Zoologische Staatssammlung München, Germany
NNE1	NEB1	1258	M	fecal	*N. nebulosa nebulosa*	UK	C	Frankfurt Zoo, Germany
NNE2	NEB6	Klah	F	blood	*N. nebulosa nebulosa*	Cambodia	W	WildAid Cambodia, Cambodia
NNE3	NEB6	-	UNK	hide	*N. nebulosa nebulosa*	Cambodia	W	WildAid Cambodia, Cambodia
NNE4	NEB1	15294	F	hide	*N. nebulosa nebulosa*	Thailand	W	Staatliches Museum für Naturkunde Stuttgart, Germany
PPA1	X	1000	M	blood	*Panthera pardus*	unknown	C	Duisburg Zoo, Germany
FCA1	X	-	F	blood	*Felis catus*	Germany	DB	Schneidemann, Tierärztliche Klinik, Würzburg, Germany
FCA2	X	-	F	blood	*Felis catus*	Germany	DB	Schneidemann, Tierärztliche Klinik, Würzburg, Germany

^a ^Identification number of individuals as they are listed at the University of Würzburg.

^b ^MtDNA assigned to each sample sequenced in this study: X not included in mtDNA analysis.

^c ^Sex of each individual: M, male; F, female; UNK, unknown.

^d ^Birth status of each individual: W, wild-born; C, captive-born; DB, domestic breed.

**Figure 2:**
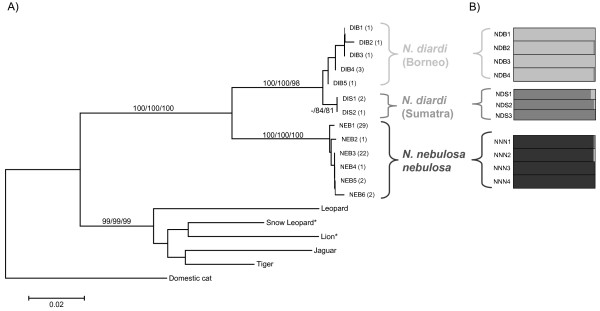
**Phylogenetic relationships among clouded leopards from mtDNA haplotypes and a Bayesian analysis of microsatellite genotypes**. (A) Phylogenetic relationships based on minimum evolution (ME) among the clouded leopard mtDNA haplotypes from the concatenated 900 bp mitochondrial sequences comprising *Cyt-b *(286 bp), *ATPase-*8 (186 bp) and control region (426 bp) gene fragments. *Panthera *samples and *Felis catus *samples were taken as outgroups. Trees constructed with PAUP [56] obtained under maximum parsimony (MP) and maximum likelihood (ML) criteria have identical topologies. Numbers above the branches represent bootstrap support (1000 replicates) for each three methods (ME/MP/ML); only those with > 80 % are shown. Numbers in parentheses represent the number of individuals sharing the same haplotype. We used Kimura 2-parameter distance with neighbor-joining (NJ) algorithm followed by tree-bisection reconnection branch-swapping procedure (TBR) for the ME analysis. MP trees were constructed using a heuristic search, with a random addition of taxa and TBR branch swapping. The ML approach was performed using a HKY85 model [60]. Haplotype codes are shown in Table 1. NEB 1 – 5, DIB 1 and 2, and outgroups have been described previously [15]. * Sequences of only two mtDNA genes (*ATPase-8 *and *Cyt b*) were included. (B) Greyscale bars are from the Bayesian admixture analysis of the microsatellite analysis. Different colours stand for different genetic groups. ID codes in are shown in Table 1.

Phylogenetic analysis of mtDNA haplotypes using minimum evolution (ME), maximum parsimony (MP) and maximum likelihood (ML) approaches produced congruent topologies that strongly support the reciprocally monophyletic status of *N.nebulosa *and *N. diardi *with high bootstrap values (100 % ME/MP, 98 % ML) (Figure 2A). Furthermore, Sumatran individuals were separated from Bornean clouded leopards supported by high bootstrap values; except for the ME approach (46% ME, 85 % MP, and 81 % ML).

A nuclear pseudogene insertion of cytoplasmic mtDNA often referred as *numt *have been reported in numerous organisms [reviewed in [18]] including the *Panthera *genus and the domestic cat [19-21]. This can complicate the analysis of mtDNA, because *numt *can coamplify with the mitochondrial genes. We cannot exclude the presence of *numts *in clouded leopards. However, it is very unlikely that we sequenced *numt *instead of mtDNA, because all our protein coding sequences had open reading frames [22]. Furthermore other nuclear genes did not amplify with our ancient DNA, even if we tried to sequence very short fragments. Therefore, we conclude that most likely we did not encounter any evidence of *numt*.

### Microsatellite analysis

Composite genotypes from 18 felid-specific microsatellite loci [23,24] were obtained from 11 clouded leopard samples (four mainland specimens NNE 1 – 4, four Bornean specimens NDB 1 – 4 and three Sumatran specimens NDS 1 – 3), two domestic cats (*Felis catus*; FCA 1 – 2) and one leopard (PPA 1). Of the 10 loci, which were used before in clouded leopards, Buckley-Beason *et al*. [15] showed that six microsatellite loci (FCA 82, FCA 105, FCA 132, FCA 144, FCA 261, FCA 310) had non-overlapping allele sizes between *N. nebulosa *(mainland) and *N. diardi *(Borneo). For only one of these six loci, FCA 261, non-overlapping allele-sizes could not be confirmed by wider sampling of Bornean and Sumatran specimens. Out of eight microsatellite loci, which were not tested in clouded leopards before, three (FCA 23, FCA 43 and HDZ 859) did not overlap in allele sizes. Overall expected heterozygosity *H*_*E *_in clouded leopards ranged from 0.488 in the Bornean population to 0.652 in *N. nebulosa *and exceeds the observed heterozygosity *H*_*0 *_in all three populations, with Bornean specimens having the lowest observed heterozygosity *H*_*0 *_with 0.361 (Table 3).

**Table 3:** Measure of genetic variance among 18 microsatellite loci in clouded leopards.

Species	N	Loci Typed	Expected heterozygosity ± SD	Observed heterozygosity ± SD	Average number of alleles/loci ± SD
***Neofelis sp***.	11	18	0.743 ± 0.125	0.439 ± 0.223	6.056 ± 1.811
*Neofelis nebulosa*	4	16	0.651 ± 0.277	0.542 ± 0.292	3.722 ± 1.626
*Neofelis diardi*	7	16	0.56 ± 0.252	0.378 ± 0.253	3.556 ± 1.383
*N. diardi *(Borneo)	4	16	0.488 ± 0.241	0.361 ± 0.291	2.556 ± 0.896
*N. diardi *(Sumatra)	3	15	0.493 ± 0.267	0.407 ± 0.343	2.278 ± 0.870

**Figure 3:**
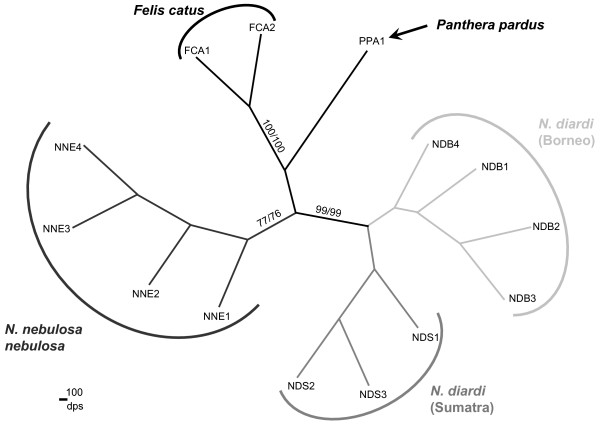
**Phylogenetic relationships among the individual clouded leopards from composite microsatellite genotypes of 18 loci**. One *Panthera pardus *sample and two *Felis catus *samples were included as outgroups. Branches of the same greyscale represent individuals from the same geographical region. Trees are based on the proportion of shared alleles (Dps) and kinship coefficient (Dkf) genetic distances with 1 – (kf/ps) option in MICROSAT [65] produced identical topologies. Dps tree is shown here. Bootstrap values over 70 % are shown on the divergence node (Dps/Dkf). ID codes are shown in Table 1.

Neighbor-joining analysis of individual clouded leopard genotypes based on two microsatellite genetic distance estimators (Dps & Dkf) produced concordant topologies; both trees support the species distinction among clouded leopards (Figure 3). Individuals from Borneo and Sumatra form a monophyletic clade with 100 % (Dps & Dkf) bootstrap support (BS) distinguishing them from mainland specimens and the outgroups. The microsatellite analysis lends further support to the phylogeographic subdivision observed in mtDNA analysis between Borneo and Sumatra individuals, however with lower bootstrap support values (Sumatra clade with 69 % Dps/61%Dkf BS, and Borneo clade with 50/46 % BS).

### Population substructures

To evaluate population distinctiveness we tested the microsatellite data using a Bayesian algorithm as implemented in BAPS version 4.14 [25]. To estimate hidden substructures, within clouded leopards BAPS suggested to partition clouded leopard into five populations (p > 0.99) three different mainland populations, Sumatra and Borneo. However mtDNA results and previous analysis based on a broader sampling, including more microsatellite loci found no substructure among mainland populations [15]. Therefore we assume that the population partition within our four mainland individuals is a result of the small sampling size and thus set a maximum of three populations. BAPS then grouped all mainland, Sumatra, and Borneo individuals together, respectively (p = 1). We confirmed the individual assignments to each population with admixture analysis in BAPS [26,27]. In this scenario all individuals were assigned to three unique clusters *N. nebulosa*,*N. diardi *(Borneo) and *N. diardi *(Sumatra) with very high admixture coefficients (all *q *> 0.98, except for one Sumatran individual (NDS1) *q *> 0.95) (Figure 2B). No individual had a Bayesian p-value less than 0.05 (all p > 0.91) revealing no evidence of admixed background and none or very low degrees of past and present gene flow between all three populations.

MtDNA sequence differences were used to estimate the divergence time of *N.nebulosa *and *N. diardi *and the time of origin of Sumatran clouded leopards. Using a calibration of 6.37 MYA for the divergence of clouded leopards from the *Panthera *lineage [14], *N. diardi *diverged from *N. nebulosa *about 2.86 MYA (95 % CI of 1.71–4.02 MYA) and Sumatran and Bornean clouded leopards diverged about 437,000 years ago (95 % CI of 30,000 – 845,000 years ago).

## Discussion

Our results strongly support the reclassification of extant clouded leopards into two distinct species *N. nebulosa *and *N. diardi*. Based on mtDNA data clouded leopards on the islands of Sumatra and Borneo have been reproductively isolated from the mainland species since middle to late Pliocene (~2.86 MYA). We are aware that recent studies showed that mtDNA is least robust in node resolution [14], which might lead to an overestimate of our calculated length of time. However previously estimated divergence time of 1.41 MYA between *N. nebulosa *and *N. diardi *[15] is still within the same range (1 – 3 MYA) as species level distinctions across *Panthera *[14]. The wider sampling of Bornean individuals dispels the doubt that the high distinction between *N. nebulosa *and *N. diardi *described before was a consequence of an inadequate sample size [15]. Referring to their origin on two Sunda Islands, we would propose to give *N. diardi *the common name "Sundaland clouded leopard".

**Figure 4:**
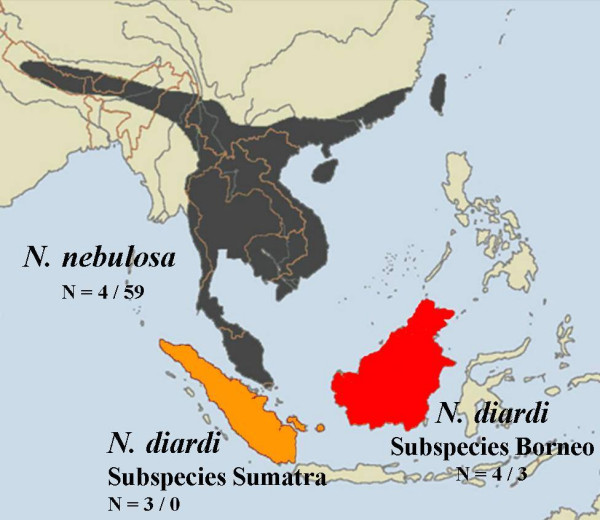
**Sampling locations and suggested new classification of clouded leopards**. The new classification is based on this molecular analysis and data obtained from Buckley-Beason *et al*. [15]. Numbers before the slash indicate the number of samples from this study, those after the slash indicate the number of samples that were included in the mtDNA analysis obtained from Buckley-Beason *et al*. [15].

Furthermore, the consistent results of mtDNA and microsatellite data provide an evidence for a reduced gene flow between Sumatra and Borneo. The trend in the reduction of observed heterozygosity might indicate a Wahlund effect, within *Neofelis*, as well as within *N. diardi*. Due to the low number of samples from each population within the sampling this result should be considered as preliminary, although it supports a hypothesised disconnection of gene flow between clouded leopard populations on the islands of Borneo and Sumatra. On the basis of our genetic results we recommend the recognition of two distinct subspecies of *N. diardi *(Figure 4), considering previous criteria for the designation of subspecies [28,29]. We estimate that since the middle to late Pleistocene Bornean and Sumatran clouded leopards were most likely isolated from each other and unable to move freely between islands. However, it has to be considered that our sample size of Sumatran specimens is small and further molecular genetic and morphological studies will be needed to confirm our findings. If these studies will support our data, we suggest giving clouded leopards on Borneo and Sumatra the scientific names *N. diardi borneensis *and *N.diardi sumatrensis*, referring to their origin.

Our results have several implications for the conservation and management strategies for modern clouded leopards. *N. nebulosa *and *N. diardi *should be managed separately, and treated as different species as suggested before [15,16]. Furthermore, on the basis of the population division between Bornean and Sumatran clouded leopards we suggest that these populations should also be managed separately. Considering previous criteria for the designation of evolutionarily significant units (ESU) [30-35], Bornean and Sumatran clouded leopards should be treated as different conservation units with separate management plans. Both populations are on the one hand reproductively isolated from each other and monophyletic for mtDNA and microsatellites and on the other hand Bornean and Sumatran individuals represent an important component of the evolutionary legacy of the species. To our knowledge there have been no translocations between clouded leopards from Borneo and Sumatra so far.

The continued depletion of tropical rainforests and fragmentation of natural habitats in Borneo and Sumatra put the reclassified species *N diardi *under severe pressure of extinction [7,36]. Therefore a higher priority should be placed on effective conservation of Sundaland clouded leopards and their shrinking habitats. Further research is urgently needed to reveal the distribution and status of different species *in situ*, because smaller distribution ranges associated with reduced gene pools of the reclassified species puts clouded leopards under a greater risk of extinction. The current IUCN Red Data category "vulnerable" [37] might underestimate the threat these cats are facing. To date only for Sabah (north-eastern part of Borneo) preliminary rough population estimates exist, demonstrating the severe threat of Bornean clouded leopards [7].

The inclusion of Sumatran samples reveals a very interesting aspect of clouded leopards' evolutionary history. At present, shallow seas separate Sumatra, Borneo, Java and Malay Peninsula. These areas were connected several times via land bridges during periods of low sea levels in the late Pliocene and Pleistocene [e. g. [38-40]]. Therefore many authors have characterized Sundaland as a geographical unit across which species should have been able to move freely during glacial periods until 10,000 years ago when higher sea-levels started to separate the islands [41-44]. Based on the presented data we cannot support the hypothesis of one geographical unit for the clouded leopard, because Bornean and Sumatran animals were reproductively isolated from the mainland individuals even during glaciation periods with accompanying low sea levels and postulated land bridges. In contrast, clouded leopards were only able to move throughout the exposed shelf between Borneo and Sumatra at least once during the early or middle Pleistocene. Therefore, despite the late Pleistocene existence of land connections [39,40], present-day distribution patterns, exemplarily shown here for the clouded leopard, indicate that dispersal was restricted and there appear to have been considerable barriers for animal migrations [40,45,46]. The wider sampling of this study in addition to previous studies [15,16] have provided a better insight into phylogeographic history of one of the least known cat species in South-East Asia.

## Conclusion

The results we present in this paper are of importance for the understanding of clouded leopard phylogeny and for their *in situ *and *ex situ *conservation and management. The different species *N. nebulosa *and *N. diardi*, as well as the two distinct populations on Borneo and Sumatra, should be managed separately, to protect the genetic diversity upon which future evolutionary potential depends. Our paper gives a good example of the importance of taxonomic splitting for conservation, because the two species and the distinct populations on Borneo and Sumatra face a much greater risk of extinction, due to smaller distribution ranges, than previously assessed based on the former classification.

The wider genetic sampling contributed to the understanding of the evolutionary history of clouded leopards. The long isolation of *N. diardi *and *N. nebulosa *revealed that the clouded leopard, comparable to other forest-dwelling species [47-51], had a deep history of vicariant evolution. Our results show that geographical or ecological barriers must have existed during Pleistocene glaciation periods hindering migrations of clouded leopards. This raises the question about the phylogeographic history of other taxa in the Sunda shelf and further research will be needed for a better understanding of evolutionary processes in this region

## Methods

### Samples and DNA extraction

We sampled four wild-born specimens from the islands of Borneo, three ancient samples from Sumatra and four recent animals from known geographic origin on the mainland (Table 1) in addition to 58 clouded leopard individuals described previously [15]. *Panthera *species and domestic cat individuals as outgroups for mitochondrial DNA (mtDNA) analysis were specified before [15] and for outgroup comparison of the microsatellite analysis one leopard (*Panthera pardus*) and two domestic cats (*Felis catus*) were sampled (Table 1).

DNA was extracted from fecal samples using QiAmp Stool Mini Kit (Qiagen, Hilden, Germany) and from serum and whole blood using QiAmp DNeasy (Qiagen, Hilden, Germany). For the extraction of DNA from historical museum samples hide and dry tissue samples were cut into small pieces using a sterile scalpel followed by a standard proteinase K digestion with an extended incubation interval at 56°C for up to 72 hours [40] until only little solid tissue remained. A standard phenol/chloroform extraction procedure was then used for DNA extraction [52]. DNA was suspended in 50 μl of double distilled H_2_O.

### Mitochondrial DNA analysis

We used a 426 bp portion of a central conserved region within the D-loop of the control region [53] in addition to two mtDNA (*ATPase-*8 and *Cyt-b*) genes [15]. The control region primers were modified by Janecka JE (pers. comm.) [(F) CTC AAC TAT CCG AAA GAG CTT] and [(R) CCT GTG GAA CAT TAG GAA TT]. In total we amplified 900 bp of mtDNA.

PCR reactions were performed in a final volume of 25 μl containing 2.5 μl MolTaq 10x PCR Buffer, 2 mM MgCl_2_, 0.2 mM dNTPs, 1 μM of each primer, 2 units of MolTaq polymerase (Molzym GmbH, Bremen, Germany) and 2 μl of genomic DNA. PCR reactions were performed in an Eppendorf Mastercycler (Eppendorf GmbH, Wesseling-Berzdorf, Germany), with an initial denaturation step at 95°C for 3 min, followed by 35 cycles of denaturation at 94°C for 30 s, annealing at 56°C for 45 s, elongation at 72°C for 45 s, and were completed with a final elongation step at 72°C for 10 min. PCR products were purified by ultra filtration through Montage TM filter devices (Millipore GmbH, Schwalbach, Germany) and sent to Seqlab (Seqlab Laboratories, Göttingen, Germany) for sequencing. Sequences were edited, assembled and aligned using ClustalW [54] implemented in BioEdit (Version 7.0.5.2) [55], before being exported to PAUP (Version 4.0b10) [56] for phylogenetic analysis. Sequences from each of the mtDNA fragments were concatenated into a 900 bp sequences, because results from separate analysis of each gene fragment showed identical topologies [57] and mitochondrial genes usually do not recombine [58]. Phylogenetic relationships among haplotypes were estimated using minimum evolution (ME), maximum likelihood (ML) and maximum parsimony (MP) [56,59]. We used Kimura 2-parameter distance with neighbor-joining (NJ) algorithm followed by tree-bisection reconnection branch-swapping procedure (TBR) for the ME analysis. MP trees were conducted using a heuristic search, with 10 random taxon addition replicates and TBR branch swapping. The ML approach was performed using the HKY85 model [60]. Each phylogenetic tree was rooted with the domestic cat sequence. Reliability of all trees was tested with bootstrap values by 1000 replicates of heuristic search and TBR branch swapping.

The approximate age of separation between *N. nebulosa *and *N. diardi *was estimated using LINTREE [61]. A neighbor-joining tree [59] was generated with Kimura 2-parameter γ-corrected distances [62] using the combined 900 bp mtDNA sequence. The molecular clock test implemented in LINTREE [61] showed that the sequences did not deviate significantly from the rate constancy test (p > 0.05). The coalescence point between clouded leopards and the *Panthera *genus, being 6.37 MYA based upon a comprehensive analysis of nuclear gene sequences and multiple fossil dates [14], was chosen to be the calibration point for this study. We used a range of two standard errors to calculate a 95 % confidence interval.

### Microsatellite markers

We used 10 felid dinucleotide microsatellite primers (FCA 8, FCA 45, FCA 77, FCA82, FCA 105, FCA 126, FCA 132, FCA 144, FCA 261, FCA 310) [23], which were already used in a previous study on clouded leopards [15]. Those microsatellites are located on 8 felid autosomes and all of them are at least 5 centimorgans apart from each other [23]. In addition to those ten microsatellite loci of known allele size ranges for clouded leopards, we applied 8 microsatellites of unknown allele sizes for clouded leopards, FCA 23 and FCA 43 [23], and HDZ 3, HDZ 57, HDZ 64, HDZ 89, HDZ817, HDZ 859 [24]. PCR amplifications were performed in a final reaction volume of 10 μl utilizing described methods [23,24]. The IR-dye-labeled PCR products were diluted and analyzed on a LI-COR 4300 DNA-Analyser (LI-COR Bioscience GmbH, Bad Homburg, Germany). Data were collected and analyzed using Saga Generation 2 (Version 3.2.1).

To test their performance as population genetic markers, all microsatellites were tested for deviations from linkage disequilibrium (LD) using GENEPOP on the web version 3.4 [63]. Measures of microsatellite genetic variation in terms of observed and expected heterozygosities were estimated with Arlequin 3.1 [64].

Pairwise genetic distance among clouded leopards and the two outgroup species was estimated with two microsatellite genetic distance estimators: the proportion of shared alleles (Dps) and the kinship coefficient (Dkf) with the [1 – ps/kf] option in MICROSAT [65]. Phylogenetic NJ-trees were constructed from the Dps and Dkf distance matrixes using NEIGHBOR (included in PHYLIP version 3.66) [66]. Bootstrap values for 1000 bootstrap replicates in MICROSAT were calculated using CONSENSE TREE (included in PHYLIP version 3.66) [66]. Trees were drawn using the program TREEVIEW (version 1.6.6) [67].

### Population structure analysis

A Bayesian clustering method as implemented in BAPS [25-27] was used to infer population structure based on multilocus microsatellite genotype data. This program estimates the hidden population substructure by testing whether the allele frequencies between populations are significantly different. A major advantage compared to most other methods is that the number of populations is treated here as an unknown parameter that can be estimated from the dataset. We performed 10 independent runs of *clustering of individuals *with the microsatellite genotypes to ensure homogenous results. In all ten runs we obtained similar results (data not shown). After the clustering of individuals by their allele frequencies the results were used to perform an admixture analysis. We used 500 iterations and a number of 1000 reference individuals per population each with 20 iterations. The estimated admixture coefficient for an individual in each cluster *q *(maximum = 1) was used as a measure of correct assignments. The Bayesian p-value tells the proportion of reference individuals simulated from the population in which the individual was originally clustered having the admixture coefficient to the cluster smaller than or equal to the individual [27]. Individuals having *p*-values larger than 0.05 are by default considered as having "non-significant" evidence for admixture [27].

## Competing interests

The author(s) declare that they have no competing interests.

## Authors' contributions

AW collected the samples for this study and carried out the molecular work at the University of Würzburg including genotyping of microsatellite markers, and mtDNA sequencing. AW also performed all sequence and microsatellite analysis and drafted the manuscript. VABB carried out the molecular work at the National Cancer Institute as well as gave very valuable comments on the manuscript. HF mentored the study at the University of Würzburg and helped to analyse the data. HF also has contributed to the preparation of the manuscript. JG participated in the study design. SJO and KEL coordinated the study at the National Cancer Institute and at the University of Würzburg, respectively, and contributed to the final revisions of the manuscript.

All authors read and approved the final manuscript.
